# Organisational health literacy in healthcare in Europe: a content analysis to explore stakeholders perspectives for supporting the adoption of effective strategies and interventions

**DOI:** 10.1186/s13690-026-01956-6

**Published:** 2026-05-16

**Authors:** Roberta Bevilacqua, Arianna Sgolastra, Cinzia Giammarchi, Hanne Søberg Finbråten, Øystein Guttersrud, Christopher Le, Denise Schütze, Julia Eder, Roberta Papa, Giulia Franceschini, Yuliia Arabska, Kateryna Marushko, Dóra Urbán-Pap, Youssoufa Ousseine, Doriane Ranaivoharison, Letizia Ferrara, Davide Montini, Study group OHL of the JA PreventNCD, Elvira Maranesi, Marco Pompili, Fabio Filippetti, Yuliia Kotykovych, Hélène Vandevalle, Christa Straßmayr

**Affiliations:** 1 IRCCS INRCA, Via Santa Margherita 5, Ancona, 60124 Italy; 2 Department of Social Determinants of Health, Norwegian Directorate of Health, Vitaminveien 4, Oslo, N-0485 Norway; 3 Department of Health and Nursing Sciences, Faculty of Social and Health Sciences, University of Inland Norway, Hamarveien 112, Elverum, N-2406 Norway; 4 The Faculty of Mathematics and Natural Sciences, The Norwegian Centre for Science Education, University of Oslo, Postboks 1106 Blindern, N-0317 Oslo, Norway; 5 Department of Community Health, Norwegian Directorate of Health, Vitaminveien 4, Oslo, N-0485 Norway; 6 Austrian National Public Health Institute, Stubenring 6, Vienna, 1010 Austria; 7 Administrative Healthcare Databases and Regional Health System Monitoring Sector, Regional Health Agency, Marche Region, Ancona, Italy; 8 Public Health Center of the Ministry of Health of Ukraine, Kyiv, Ukraine; 9 National Center for Public Health and Pharmacy, Budapest, Hungary; 10 Prevention Department, French National Cancer Institute, 52 Avenue André Morizet, Boulogne-Billancourt, 92100 France

**Keywords:** JA PreventNCD, Organisational health literacy, Healthcare organisations, Policies, Interventions, Strategies, Stakeholders involvement, Assessment, Multidisciplinary approach, Public health

## Abstract

**Background:**

Organisational Health Literacy (OHL) refers to the capacity of healthcare organisations to enable people to find, understand, appraise, and use health information and services equitably. Although interest in OHL has grown across Europe, the degree to which healthcare organisations adopt and assess OHL remains uneven.

**Objective:**

This study explored stakeholders’ perspectives on factors that facilitate or hinder the uptake of OHL strategies and assessments within healthcare organisations in Europe.

**Methods:**

Semi‑structured interviews and surveys were conducted with stakeholders from six European countries within the JA PreventNCD initiative. Data were analysed using qualitative content analysis to identify macro‑ and micro‑themes related to OHL adoption and assessment. A SWOT analysis was conducted to provide a framework for discussing the factors that facilitate and hinder the adoption of OHL in healthcare organisations.

**Results:**

Across countries, stakeholders highlighted several overarching themes: the importance of leadership commitment, staff training, and integrating OHL into organisational processes; the need to involve patients and communities; and key strategies to support OHL assessments—communicating their added value, securing managerial or authority‑level incentives, and using practical tools. A lack of awareness of OHL, scarce resources and management support, and overly complex tools, actors and steps to secure commitment were also identified as issues to address.

**Conclusions:**

Stakeholders across diverse health systems emphasised that strengthening OHL requires coordinated organisational action rather than isolated activities. Prioritising leadership engagement, embedding OHL into quality improvement structures, and supporting staff through training and accessible tools are crucial to creating sustainable OHL‑responsive environments. These findings provide actionable insights for designing and implementing OHL strategies and assessments in European healthcare organisations considering facilitating and hindering factors for their adoption.

**Supplementary Information:**

The online version contains supplementary material available at 10.1186/s13690-026-01956-6.

**Table:** 

Text box 1. Contributions to the literature
• Despite the recent interest in OHL throughout Europe, it is not yet well integrated into healthcare organisations. This is characterised by a lack of clear strategies, evaluation and monitoring
• The promotion of OHL in healthcare organisations across Europe is dependent on an exploration of the factors that either facilitate or hinder this process involving stakeholders
• The analysis of stakeholders' interviews has resulted in the emergence of
• Their perspective on OHL and its adoption in six European countries—Austria, France, Hungary, Italy, Norway, and Ukraine. This provides a starting point for the planning of future actions

## Background

Information is a crucial determinant of health outcomes, and organisational health literacy (OHL) provides equitable access to health information and resources for all. OHL is defined as the extent to which organisations facilitate equitable access for people, through organisational structures, policies and processes, to find, understand, appraise and use information and services to inform health-related decisions and actions for themselves and others [1, 2]. Users, staff and community residents can benefit from enhanced OHL [2, 3]. Specifically, organisations should engage all stakeholders (patients, relatives, staff, leaders and citizens) to make informed judgements and decisions regarding healthcare, disease prevention and health promotion to maintain or enhance their quality of life [1].

By promoting OHL, organisations can directly influence people’s health and well-being, thereby reducing health inequalities. Insufficient individual health literacy (HL) has been associated with risk behaviours (e.g., poor diet and smoking) [4, 5], a lower capacity to manage social stress [6], and less adherence to treatments [7, 8], all of which can contribute to an increased use of health services, less effective prevention, and worse long-term outcomes [7–11]. Therefore, health organisations should have strategies that facilitate access to, understanding of, and the use of information and health services, effectively meeting the HL needs of all stakeholders [5–12]. Recent reviews indicate that OHL interventions have a positive impact on patient-related outcomes, including improvements in HL skills, greater engagement in healthcare, and enhanced self-management capabilities [13, 14]. In addition, OHL has been identified as a factor influencing patients' satisfaction [15].

Bremer et al. [16] described six criteria and attributes of a health-literate organisation: (i) ‘communication with service users’, (ii) ‘easy access and navigation’, (iii) ‘integration and prioritisation of OHL’, (iv) ‘assessment and organisational development’, (v) ‘engagement and support of service users’, and (vi) ‘information and qualifications of staff’.

In practice, a health-literate organisation adopts HL best practices in all its structures and processes.

A health-literate healthcare organisation actively engages stakeholders in the development of documents, materials and services to ensure that they are clear and accessible. It also invests in staff training to strengthen competencies at both the individual and organisational levels and adopts effective navigation strategies [17]. This multidimensional perspective can be visualised using a matrix that links HL domains with key stakeholder groups, helping organisations to identify where to intervene and how to align strategies across sectors and functions. For example, while ‘clear communication’ affects both patients and staff, ‘staff training and competencies’ are primarily linked to internal capacity building, and ‘post-discharge support’ directly involves patients and families. The use of such a matrix facilitates a holistic view of OHL implementation, moving beyond individual actions to promote integrated, sustainable change throughout the organisation [17].

Therefore, to apply the OHL concept in a systematic and sustainable manner, a healthcare organisation will have to use the principles and tools of quality management, as well as promoting and building specific organisational capacities to become more health-literate [1].

Research highlights the development and validation of various tools developed to measure and improve OHL in multidisciplinary settings. In healthcare settings, self-assessment tools are used, including the International Self-Assessment Tool for Organisational Health Literacy of Hospitals (OHL-Hos) [17–19] and its short version (OHL-Hos-SF) [20], the International Self-Assessment Tool for Organisational Health Literacy in Primary Health Care Services (OHL-PHC) [3] and the Health-Literate Healthcare Organisation-10 items questionnaire (HLHO-10) [21, 22].

Over the past few years, HL and OHL have received increasing attention in Europe, as they are recognised as crucial factors in improving health outcomes and promoting equity in access to health services.

In the Joint Action Prevent Non-Communicable Diseases (JA PreventNCD), a European Joint Action [23], a mapping exercise was carried out in 2024 [24], to explore the literature and national information, materials, tools and activities on OHL in healthcare organisations in six countries.

For the mapping, an ad hoc template was developed to explore key issues relevant to understanding the national context of OHL and was refined based on feedback from the participating partners. The areas of interest were informed by the checklist presented in *Health literacy policies – how can they be developed and implemented? A guide for policy and decision makers* [25]*.* The final template covered seven main areas: (1) policy framework supporting OHL responsiveness in health care services, (2) current state of national OHL in health care services, (3) tools and supportive documents/materials on OHL, (4) interventions, (5) evaluation and monitoring, (6) cooperation and (7) research. For each area, specific questions were defined to specify the content to be mapped.

The mapping suggested that OHL is not well established within healthcare organisations in the participating countries and is characterised by a lack of clear strategies, evaluation and monitoring.

Although progress has been made in adopting interventions and policies to enhance HL and OHL in Europe, significant challenges remain [13–26]. These include a direct inclusion or mentions of OHL in policies, strategies or specific actions and, when the direct reference to OHL is considered, it remains a marginal issue. There is a need to improve advocacy and stakeholders’ engagement, supporting documents and materials in national languages, enhance strategies to support OHL, take in to account OHL in accreditation system of healthcare services, improve best practice and integrate OHL into evaluation and monitoring. It is therefore necessary to investigate the perceptions and experiences of relevant stakeholders within healthcare systems regarding OHL, in order to better understand existing hindering factors, opportunities for improvement and possible actions to strengthen OHL. In detail, recognising and understanding the specifics of these challenges could be useful for creating a foundation for intervention tailored to OHL adoption, moreover, since OHL is a multidisciplinary issue, it is also essential to engage diverse stakeholders to tailor OHL interventions to the specific needs of individuals, organisations, and national contexts.

In this context, the key objective of this study was to explore facilitating and hindering factors for promoting the adoption of OHL and its assessment healthcare organisations in Europe.

## Methods

### Design

The study has a qualitative design and was performed by research teams from six European countries —Austria, France, Hungary, Italy, Norway, and Ukraine — involved in the framework of the JA PreventNCD [23, 24]. This exploratory study employed content analysis on data from semi-structured interviews with relevant stakeholders from the participating countries. The mapping was conducted in preparation for the development of a tool to assess the OHL of hospitals and primary care facilities.

### Selection of stakeholders

The research teams agreed on the criteria for identifying relevant stakeholders. These were: (1) stakeholder should have a demonstrated expertise in OHL in the participating country, specifically through their professional experience, in facilitating factors that enhance OHL, in supporting the implementation and/or uptake of OHL assessment tools within healthcare organisations and (2) insights into the promotion and/or assessment of OHL in hospitals or primary care services. Ideally, the stakeholder should hold a position that involves facilitating, promoting, implementing, or assessing OHL within healthcare organisations.

A total of 83 stakeholders were selected for this study. Relevant stakeholders included experts in OHL or HL, experts in OHL and NCD and/or cancer prevention, healthcare policy makers, managers or staff involved in OHL assessments, managers or staff who opted not to participate in OHL assessments, healthcare organisation leaders, staff in influential roles within healthcare services, representatives from professional associations (e.g. medical associations) and patient representatives.

The number of stakeholders interviewed were: six in Austria, ten in France, nine in Hungary, eleven in Italy, forty in Norway and seven in Ukraine. The stakeholders were predominantly female, although gender distribution varied across countries and this information wasn’t available for all the countries. Age information was available for the Italian and Ukrainian stakeholders, ranging from 33 to 75 years. Additional details of the sociodemographic characteristics and professional backgrounds of stakeholders and experts by country are provided in the Supplementary Materials.

### Data collection

A semi-structured interview guide was developed. Questions were formulated to explore the detailed research questions: how organisations can become health literacy responsive, what support or hinders OHL assessments and interventions, which organisational factors are essential for adoption, and what steps are needed for mandate and commitment. The criteria for question development ensured conceptual alignment with OHL definitions, relevance to implementation processes in hospitals and primary care, and sufficient breadth for stakeholders with OHL expertise to provide meaningful, in-depth responses. The “Results” section is structured according to the detailed research questions.

In Austria individual interviews were conducted face to face personally or online. In France nine individual interviews were held online and an interview was conducted with two stakeholders at the same time. In Hungary one interview was conducted online and eight were administered as online surveys with open-ended questions. In Italy, eleven stakeholders were initially contacted by phone to introduce the questionnaire and provide clarifications, before each respondent completed the survey online with open-ended questions independently. In Norway, data were collected during a workshop with forty stakeholders, who were divided into eight groups and each group responded to one or two questions from the semi-structured interview. In the Ukraine the individual interviews were administered as mail surveys. In the case of face-to-face interviews, the interviews were transcribed.

The interviews were anonymised in each country, and the content of the interviews was translated into English for the international analysis. Translations were conducted by the respective national research teams. The translating researchers ensured accuracy by referring back to the original interview notes or summaries when wording required clarification.

### Analysis

First, all data were synthesised using a shared-structure developed from the interview guide. The obtained textual data were divided in macro-themes into MAXQDA software [27]. Second, the macro-themes previously identified were broken down into micro-themes, which were manually coded by the evaluators, via an organic coding process. This approach enabled a detailed and structured categorisation of the content of the responses, ensuring a more in-depth interpretation in the qualitative analysis. The two evaluators separately identified micro-themes to ensure inter-rater reliability and to avoid redundancy and biases related to text comprehension. For question 4, only macro-themes were coded, since these already reflect the essential content of the responses.

Any discrepancies (including differing code assignments, theme boundaries, or interpretations) were systematically resolved through iterative peer debriefing sessions with additional research team members not involved in initial coding. In these discussions, the team reviewed original texts, compared rationales, and reached consensus by refining codes, reassigning segments, or merging/splitting themes as needed, ensuring unanimous agreement on all final outputs and high inter-rater reliability. These sessions specifically addressed discrepancies between the two coders by reviewing original texts, discussing rationales, and reaching consensus through iterative refinement of themes, with the final aim of maximizing the inter-rater reliability.

Additionally, a SWOT analysis was conducted to provide a framework for synthesising the factors that facilitate and hinder the adoption of OHL in healthcare organisations to structure the discussion of common strengths, weaknesses, opportunities and threats emerging from the results.

## Results

This section outlines the results of the qualitative analysis. For each question, the emerging macro-themes are described together with their associated micro-themes, with the intention of examining the different nuances identified in the responses and preserving the complexity and richness of the qualitatively analysed material.

### How can healthcare organisations be transformed to health literacy-responsive organisations?

**Fig. 1:**
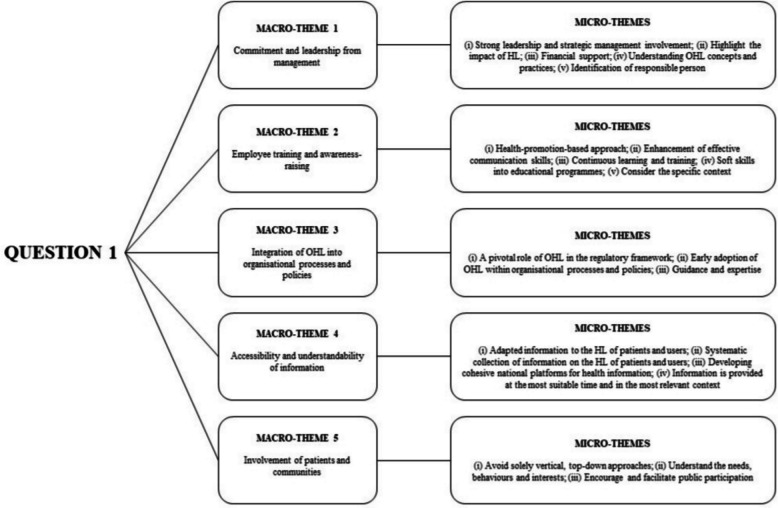
Macro- and micro-themes of question 1

A compact report of the macro and micro-themes associated to question 1 is reported in Fig. 1.

#### Commitment and leadership from management

From the perspective of stakeholders, healthcare organisations must adopt a comprehensive approach to enhance HL. A main factor highlighted by stakeholders was the need for strong leadership and strategic managerial commitment, which is essential for fostering a culture that prioritises HL at all levels of the organisation.

This includes the concrete commitment from leadership to promote OHL; a willingness to adopt organisational best practices; the establishment of internal systems for continuous professional development by managers; the importance of leading by example; the adoption of a top-down approach; and the recognition of the need for permission and formal support from leadership to act effectively.

Furthermore, to stimulate the adoption and prioritisation of OHL, according to stakeholders, it is crucial that the benefits are clearly perceived at both the individual and organisational levels. It is important to collect self-assessment data to *highlight the impact of OHL*, as well as to communicate and demonstrate benefits, possibly with the support of coaches and organisational development experts.

As understood by stakeholders, leadership commitment also involves providing the necessary tools and resources to promote HL and teamwork among professionals. Sufficient *financial support* for the adoption of OHL-related initiatives, including incentives for training and conducting self-assessments, is also important. *Understanding OHL concepts and practices* within organisations is also considered an essential prerequisite for the success of the entire process of adopting OHL in healthcare organisations, also with the adoption of specific measures and tools.

Another main point that emerged from stakeholders’ point of view was the *identification of responsible persons* within the organisation to facilitate the adoption of essential activities. Such persons could be from quality management, and an HL contact within each team could be appointed.

#### Employee training and awareness-raising

The stakeholders emphasised the need to educate healthcare staff about the importance of HL, emphasise communication skills, develop empathy, and tailor information to patients' needs.

Specifically, one of the main issues that has emerged is the shift from an exclusive focus on the care model to a *health-promotion-based approach*. This implies that healthcare staff training should aim to strengthen the legitimacy of health promotion approaches and recognise the impact of healthcare organisations on the broader community, thus supporting HL for all.

Another crucial topic that has emerged from the stakeholders’ replies is the *enhancement of effective communication skills* among healthcare staff. Training should focus on developing content- and form-conscious communication, providing staff with the basic skills to help patients and their families navigate the healthcare system. Improving the effectiveness of verbal communication is paramount. Moreover, poor communication negatively affects patients' HL and treatment outcomes.

The importance of *continuous learning and training* becomes clear for the stakeholders: continuous learning should not be limited to clinical skills but should also include professional HL. Ways to support continuous learning include creating Communities of Practice for the exchange of knowledge on OHL, integrating HL and health promotion in team and network meetings, and investing in internships. From the perspective of stakeholders, training should not only be aimed at doctors and nurses but also at all personnel who are in contact with patients, including administrative staff. Providing HL training at all levels is essential to make people aware of the importance of their role in ensuring the HL of citizens/patients.

The integration of *soft skills into educational programmes* for health professionals at both the undergraduate and postgraduate levels is a widely recognised issue by stakeholders. Curricula should include courses or modules emphasising HL principles, communication strategies, and cultural competence and empathy, aiming to teach how to explain complex medical information in accessible language.

Finally, as understood by stakeholders, training should *consider the specific context* in which the organisation operates to communicate effectively with patients who may have different cultural, psychological or social needs.

#### Integration of OHL into organisational processes and policies

Stakeholders in several countries indicated that activities on HL and OHL should not be treated as isolated initiatives but rather integrated into organisational policies and processes.

In particular, the countries that brought this forward assumed that there is a need for *a pivotal role of OHL in the national regulatory framework*, with a particular reference to policies and regulations relevant for organisations and healthcare professionals.

An *early adoption of OHL within organisational processes and polices,* according to stakeholders, is needed to take place during the foundation and/or opening phase of primary care organisations to avoid later change processes.

It also emerges that it is essential to develop an internal policy involving all those responsible for healthcare structures (departments, units, general and medical directors) to coordinate human and technological resources and to orient the organisation towards an improvement in OHL, thus enabling *guidance and expertise* on this issue.

#### Accessibility and understandability of information

Stakeholders agreed that ensuring the accessibility and understandability of information is a priority.

Stakeholders emphasised that health information should be *adapted to the HL of patients and users.* It is essential to help people with low HL levels navigate information, and it is advisable to use visual aids such as information boards, clear signage, easy-to-understand infographics, illustrated leaflets, and explanatory videos. These materials should be easily accessible and contain up-to-date information.

An important step that should be taken, according to stakeholders, is the *systematic collection of information on the HL of patients and users*. This allows health professionals to be more aware of the difficulties that people may have in accessing and understanding health information, as well as their literacy and digital skills. Personalising the message, considering individual characteristics and context, improves the effectiveness of communication and promotes adherence to care pathways.

The importance of *developing cohesive national platforms for health information,* such as the “HelseNorge” platform [28], where quality-verified content is available, was emphasised. The aim is to collect, using one platform, all the information patients and users need, from general medicine to specialised care via rehabilitation, dental health, mental health, physiotherapy and other services. It could be useful to expand existing systems to improve the credibility of health websites.

Finally, from stakeholders’ point of view it is essential that *information is provided at the most suitable time and in the most relevant context*. Even in the early stages of the care pathway, communication should be systematised, and information should be shared consistently. This approach facilitates patients' understanding, memorisation and effective use of information.

#### Involvement of patients and communities

The engagement of patients and communities in the development of health services is considered essential in several countries. An HL-sensitive organisation should *avoid solely vertical, top-down approaches*. Instead, it must foster participatory processes that actively involve patients, carers and communities in setting priorities and designing services.

One of the first steps in building truly useful and effective services, according to stakeholders, is to listen to and *understand the needs, behaviours and interests* of the people within the different healthcare settings. This requires a careful analysis of the specific needs of each context to identify the critical issues to be addressed and the priorities on which to build targeted and customised interventions. Understanding which issues to intervene in also means recognising the cultural, social and linguistic diversity of patients and adapting messages and communication channels accordingly.

According to the stakeholders interviewed, a truly responsive healthcare organisation should *encourage and facilitate public participation*, involving citizens (including social representatives) in decision-making processes. Organisations should create regular opportunities for listening and consultation, where people can not only express their satisfaction or dissatisfaction but also actively contribute suggestions and observations based on their own experiences. Even without technical expertise, citizens can identify needs, shortcomings and critical issues that are often invisible to decision-makers.

### What would support the adoption of OHL assessment in hospitals and primary healthcare services?

**Fig. 2:**
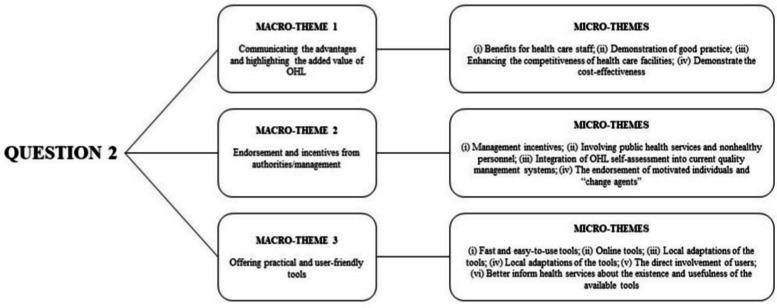
Macro- and micro-themes of question 2

A compact report of the macro and micro-themes associated to question 2 is reported in Fig. 2.

#### Communicating the advantages and highlighting the added value of OHL

The stakeholders stressed that, in order to introduce OHL assessments, it is vital to highlight their benefits and added value. They also noted the various advantages of a health literate healthcare organisation, such as increased quality of healthcare, lower costs and increased satisfaction for patients and staff. The importance of clearly communicating the advantages of participating in OHL activities was emphasised, highlighting the added value it can provide to both patients and professionals. Particular importance was placed on the *benefits for healthcare staff*, such as improved mental health and teamwork, which may be even more compelling than the economic arguments alone. Management participation is also crucial, as they must understand the advantages of using self-assessment tools. It is important to show in a tangible way the advantages that adopting OHL can bring, both in terms of organisational efficiency and quality of care. Furthermore, understanding the positive impact of OHL on patient compliance and clinical outcomes in primary care is an additional incentive for adopting OHL.

According to stakeholders, another strategic element is the *demonstration of good practice* and the use of concrete examples of success, such as the introduction of the mhGAP [29] in other institutions, to make the value of OHL more immediately perceptible. In addition, there is the possibility of *enhancing the competitiveness of healthcare facilities* by enhancing OHL as a distinctive element that can attract patients.

Finally, the potential of benchmarking by comparing results between similar facilities was underlined, as was the need to *demonstrate the cost-effectiveness* of OHL by highlighting its contribution to saving time and resources.

#### Endorsements and incentives from authorities/management

According to stakeholders in all countries, support from management and health authorities is essential, and *management incentives* play a key role in the adoption of assessment. First, the need for incentives from the authorities is emphasised: institutional support, including specific incentives, is considered crucial to promote the implementation of OHL assessment. Among the main supporting instruments, sufficient funding was mentioned as an incentive to facilitate the integration of OHL into organisational processes. Stakeholders also emphasise the importance of tailoring incentives to specific context and adapting them to different settings, such as hospitals or primary care services. Furthermore, they highlighted the need to include HL as a strategic element in economic models and service standards as well as a formal indication by regulatory bodies.

Strong impetus from management and support from department heads is needed to confirm the decisive role of leadership in actively supporting OHL assessments. The stakeholders also suggested *involving public health services and non-health personnel* in the assessment process to ensure a more balanced and inclusive approach.

Another main aspect, raised by stakeholders, concerns the *integration of OHL assessment into current quality management systems*, anchoring it firmly at the managerial level and involving, for example, quality and health promotion managers. The compatibility of OHL with existing systems is considered favourable for its adoption.

Additionally, *the endorsement of motivated individuals and “change agents”,* such as health promotion staff, nurses and social workers, who can act as catalysts in the OHL implementation process, is crucial.

#### Offering practical and user-friendly tools

Several countries have stressed the need for practical and intuitive assessment tools that are clear, adaptable, and time efficient.

First, the need for *fast and easy-to-use tools* is emphasised: tools that can be filled out quickly, require minimal resources to respond to, and are embraced by users. The importance of simplicity also extends to the understanding and interpreting the results, which should be immediate and easy-to-use.

Stakeholders stated a clear preference for *online tools* that can be accessed via QR codes, for example.

The need to provide *local adaptations of the tools* to respond to the specificities of the different healthcare contexts was also emphasised. A difference is recognised between primary care and hospital contexts: whereas in the former, self-assessment seems easier to introduce, in hospitals, the organisational complexity makes it more difficult to identify responsible persons.

The *direct involvement of users* in the creation of tools is also fundamental: rather than starting from already structured questionnaires it is recommended to begin with a more exploratory assessment of the context, needs and expectations to build a strategy for analysing practices that is truly calibrated to the reality of reference.

Another point raised by stakeholders concerns the need to *better inform health services about the existence and usefulness of the available tools,* also considering the workload of employees. In this sense, it is important for management to carefully evaluate the number of surveys and initiatives proposed to staff to avoid excessive overload. A concrete example of the usefulness of OHL tools is the OHL Action Plan implemented by a Czech hospital as result of an OHL self-assessment process [30].

### Which factor could hinder the OHL assessment in the hospitals and primary healthcare services and how could these be overcome?

**Fig. 3:**
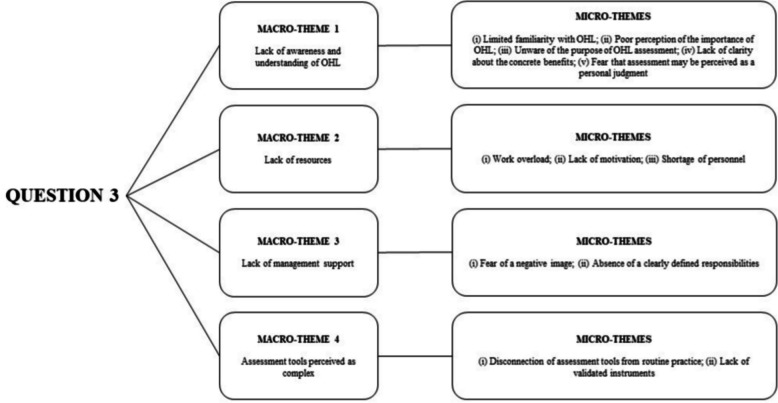
Macro- and micro-themes of question 3

A compact report of the macro and micro-themes associated to question 3 is reported in Fig. 3.

#### Lack of awareness and understanding of OHL

A significant hindering factor for OHL assessment in hospitals and primary healthcare services is the lack of awareness and understanding of OHL among staff and management.

From the stakeholders’ point of view, there is widespread *limited familiarity with OHL*, to the extent that it is difficult to see how it can be developed without first understanding what it is. This lack of awareness is particularly evident among healthcare professionals themselves, representing a substantial hindering factor, making it difficult to propose targeted solutions without a full understanding of the situation and available resources.

The *poor perception of the importance of OHL* among healthcare professionals, according to stakeholders, is a critical obstacle that can only be overcome through strong promotion and awareness initiatives from healthcare management and administration. Many practitioners are *unaware of the purpose of OHL assessment*, and the *lack of clarity about the concrete benefits* risks limiting their acceptance and adherence. This is exacerbated by a misperception of the usefulness of OHL, which is often perceived as a purely academic concept far removed from the practical needs of patients and healthcare facilities. Stakeholders reported that the benefits of assessment are not clearly perceived by those who should adopt it; without a clear short-term positive impact on daily practices, efforts risk remaining confined to a theoretical exercise. Finally, there is a *fear that assessment may be perceived as a personal judgement* of the individual practitioner rather than as a tool for improving services.

#### Lack of resources

Stakeholders highlighted *work overload* as one of the main obstacles to the implementation of OHL assessments in hospitals and primary care services. Many professionals perceive the introduction of new assessment activities as placing an additional burden on their already heavy workload. In contexts with limited resources, the adoption of new tools may be seen as a low priority. This perception of an “additional task” has been observed in several contexts and represents a significant barrier.

Another critical issue identified is the *lack of motivation*. Attitudes of resistance to change may be fuelled by difficulties related to a lack of resources, which is particularly evident in structures where a large proportion of staff are elderly or close to retirement. Moreover, if the introduction of assessment tools is perceived as an imposition from above, without a clear perceived benefit, the risk of low motivation increases further.

A *shortage of personnel* is another significant obstacle according to stakeholders, especially in small primary care facilities, where there is often a lack of specialised personnel capable of supporting assessment activities effectively. Limited human resources, in terms of both quantity and quality, make initiating and supporting self-assessment processes more complex.

#### Lack of management support

The lack of support from management further complicates the implementation process and was highlighted by all the countries. The absence of active leadership involvement was identified as a major critical issue, making it difficult to create an environment that enables the stable and structured integration of OHL assessments.

The stakeholders first identified *the fear of a negative image* among management. There was a strong concern that a negative assessment could undermine the reputation of the healthcare organisation, thus leading facilities to focus more on appearance than on improving the quality of services offered to citizens. This phenomenon has been described as a well-known effect on the functioning of organisations.

Another recurring issue raised by stakeholders concerns the *absence of clearly defined responsibilities*: it is often unclear, within healthcare organisations, who should take responsibility for promoting HL among patients.

#### Assessment tools perceived as complex

Several countries also noted that long, extensive assessment tools that are perceived as being complex, time-consuming or impractical, may discourage the application.

From the perspective of stakeholders, another set of factors that could hinder the implementation of OHL assessments in hospitals and primary care services is the perceived *disconnection of assessment tools from routine practice*. In fact, the available tools are often considered to have little bearing on the operational reality of healthcare settings. In addition, the *lack of validated tools*, specifically questionnaires designed for different target groups, is a particularly relevant issue in primary care.

### Which persons/roles in hospitals/primary healthcare services could be considered as door openers for initiating an assessment?

**Fig. 4:**
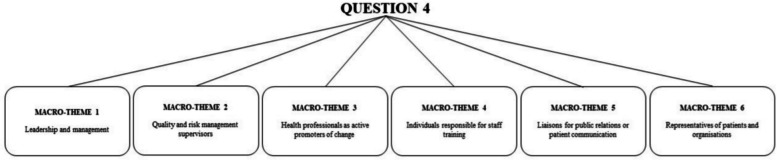
Macro- and micro-themes of question 4

A compact report of the macro and micro-themes associated to question 4 is reported in Fig. 4.

In order to introduce a process of OHL assessment within hospitals and primary care organisations it is essential, according to stakeholders, to identify those professionals who, due to their role, vision or sensitivity, can act as true ‘pathfinders’ in the process. According to the stakeholders interviewed, various professionals can play a decisive role in promoting and supporting HL-oriented organisational analysis.

First, *leadership and management*, such as general managers, department heads and facility managers, are central figures from the stakeholders’ point of view. They have the authority and strategic vision to formally initiate an OHL evaluation process and integrate it into organisational priorities.

Another key group, according to stakeholder, is *quality and risk management supervisors*. These professionals are familiar with evaluation processes and continuous improvement systems and often show interest in initiatives that enhance safety, transparency and patient-centredness—all aspects closely related to HL. Health promotion contact persons within healthcare organisations may also be naturally inclined to support OHL initiatives owing to their focus on equity, prevention and empowerment of the individual.

Among the most influential actors, raised by stakeholders, are *health professionals as active promoters of change*, affecting the organisational culture through their daily contact with users.

Another main element, according to stakeholders, is the involvement of *individuals responsible for staff training*. Training managers can demonstrate how OHL is a useful tool for enhancing the skills of health personnel, improving communication and strengthening the quality of services provided.

Additionally, *liaisons for public relations or patient communication* should not be underestimated. These professionals collect feedback, handle reports and address the information needs of users, often developing a specific sensitivity to issues of communicative accessibility.

*Representatives of patients and organisations* can also offer a significant boost as understood by stakeholders. Their active involvement enables the voice of citizens to be brought into assessment processes, helping to highlight critical issues and proposals for improvement directly from the point of view of lived experience.

### What concrete steps are necessary to obtain a mandate on OHL assessment from the responsible persons?

**Fig. 5:**
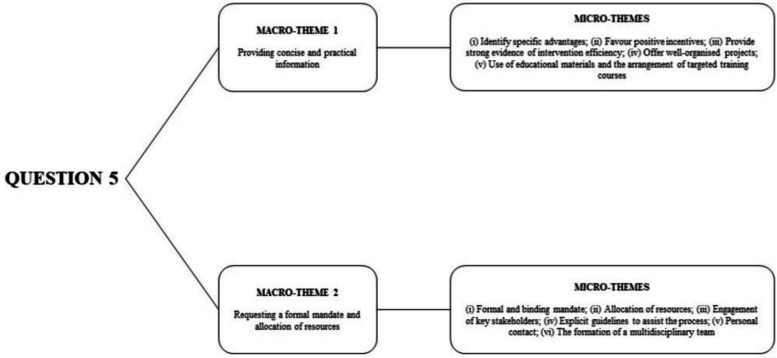
Macro- and micro-themes of question 5

A compact report of the macro and micro-themes associated to question 5 is reported in Fig. 5.

#### Providing concise and practical information

The collected responses clearly indicate that a few specific actions are required to secure a mandate for an OHL assessment. First, it is crucial to *identify specific advantages* that healthcare facilities can gain from adopting an OHL assessment, going beyond theoretical evidence and emphasising practical advantages, such as increased attractiveness to decision-makers. It is recommended to favour incentives, such as awards or recognition, rather than overly restrictive standards. Furthermore, the initiation of a national research project and the possibility of scientific publications can be additional motivational levers. Dedicated financial incentives and the adoption of simple actions with visible impact in a short amount of time are useful strategies to keep the motivation of engaging in OHL activities high.

The stakeholders also emphasised the need to *provide strong evidence of intervention efficiency*, showing how investing in HL can help reduce costs, improve the quality of services, increase user satisfaction and reduce health inequalities.

It is also important, according to stakeholders, to prepare and *offer well-organised projects* that are anchored in existing quality networks, and to adopt an operational and action-oriented approach. Another main component raised by stakeholders is the *use of educational materials and the arrangement of targeted training courses*, accompanied by appropriate certifications, taking advantage of existing events, networks and platforms to promote an OHL responsive culture, including practical tools such as webinars.

#### Requesting a formal mandate and allocation of resources

From the answers collected, it appears that, to seek and obtain a formal mandate from managers for OHL assessment, it is crucial to emphasise various specific points. First, the need for a *formal and binding mandate* that explicitly includes the assessment of OHL was emphasised, ideally within the commissioning or strategic documents of healthcare organisations. It is also important to ensure that the language used is clear and prescriptive, favouring terms such as 'must' instead of 'should' to reinforce the mandatory nature of the commitment.

In parallel, the importance of *allocating resources* was highlighted: without adequate funding, the introduction of an assessment cannot be strategic, organisational or systematic.

Another main point raised by stakeholders concerns the *engagement of key stakeholders*, such as healthcare management and ownership representatives (e.g. the local council). It is essential to obtain their commitment through direct and customised approaches.

According to stakeholders, the leaders of healthcare organisations should also provide *explicit guidelines to assist the process*, accompanied by practical examples and concrete actions to facilitate effective adoption.

Finally, the need for a practical and well-defined approach is emphasised: *personal contact with managers* is considered much more effective than email communication alone. The appointment of an operational project manager and *the formation of a multidisciplinary team* were also suggested.

### What concrete steps are necessary to obtain continuous commitment towards OHL responsiveness in healthcare services (hospitals and/or primary care services)?

**Fig. 6:**
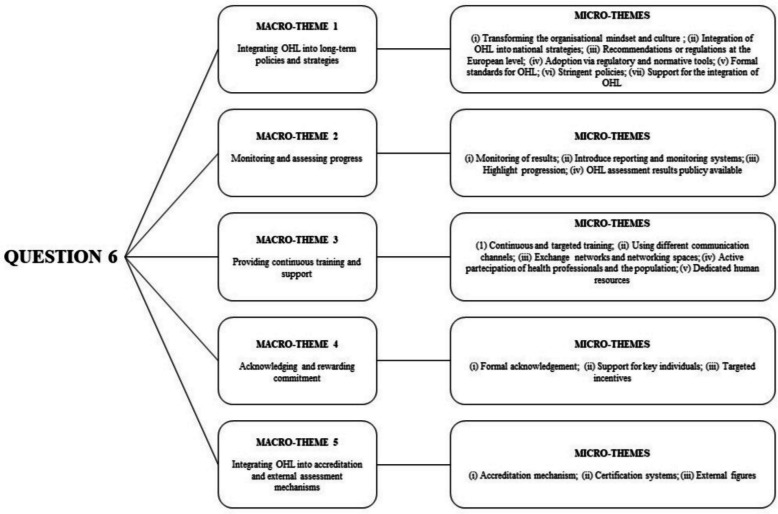
Macro- and micro-themes of question 6

A compact report of the macro and micro-themes associated to question 6 is reported in Fig. 6.

#### Integrating OHL into long-term policies and strategies

According to the stakeholders' responses, several steps are necessary to ensure a continuous commitment to OHL responsiveness in health services. The first element concerns *transforming the organisational mindset and culture.* This change must start at the top, with a clear and strongly supported mission from the leadership, accompanied by the availability of suitable resources. However, transforming the organisational culture requires time and ongoing commitment.

A second aspect raised by stakeholders concerns the *integration of OHL into national strategies*, emphasising OHL within health policies, or for a dedicated strategy to be developed. Stakeholders also proposed pushing for *recommendations or regulations at the European level* to strengthen the political and institutional focus on the topic.

Alongside those aspects, the importance of *adoption *via* regulatory and normative tools* was highlighted, with the suggestion that OHL be anchored in the health system through legal regulations, ordinances and official guidelines, as is already the case in some contexts through inclusion in quality systems and quality of care reports. The definition of *formal standards for OHL* was also suggested.

Another recurring issue is the need for more *stringent policies. A*ccording to the stakeholders, it would be appropriate to introduce legislative obligations that would make the assessment of OHL in healthcare facilities systematic.

Finally, from the perspective of stakeholders, the process of integrating OHL into long-term health strategies requires *support for the integration of OHL*, including defining and applying clear standards and practical improvement measures.

#### Monitoring and assessing progress

In order to achieve a sustained commitment to OHL in health services, stakeholders believe that a number of concrete actions must be taken. Firstly, it is crucial to ensure constant *monitoring of results.* Only through long-term action and continuous supervision will it be possible to improve interventions, respond effectively to health needs and successfully disseminate health promotion messages. Therefore, it is necessary to *introduce reporting and monitoring systems* for both the results achieved and any deviations from the objectives. Providing feedback on the results of the assessment, including data on the target population, where possible, can make the process more transparent and improvement oriented. In this sense, it is important to *highlight progression*, actively promoting the exchange of good practices, to foster mutual learning and collective growth.

Finally, the usefulness of making *OHL assessment results publicly available* was emphasised to increase transparency, facilitate comparison and recognise the efforts made.

#### Providing continuous training and support

An ongoing commitment to OHL in health services requires *continuous and targeted training* of staff, involving all employees. This training should aim at increasing awareness of the importance of HL as a training tool, as well as improving relationships with patients and society. It is therefore necessary to organise specific courses also on the practical introduction of OHL at the organisational and clinical levels, ensuring regular updates.

At the same time, stakeholders stressed the importance of *using different communication channels* to inform patients, enhancing existing ones and developing new ones with the support of dedicated professionals. The production of centralised information materials, such as leaflets or interviews, intended for both healthcare professionals and patients, represents concrete support.

An additional strategic measure is to establish local and national *exchange networks and networking spaces* dedicated to OHL. To consolidate this approach, it is recommended by stakeholders that OHL be integrated into corporate training plans through multiyear development programs and the organisation of regular meetings in all operational areas, as well as the proposal to dedicate a national conference dedicated exclusively to this topic.

The *active participation of health professionals and the population*, for example by setting up focus groups, is seen as an important means of dissemination and strengthening public relations.

*Dedicated human resources* are also indispensable according to stakeholders. The need to appoint a contact person is emphasised, possibly through the creation of a permanent position with specific resources allocated for this purpose.

#### Acknowledging and rewarding commitment

Stakeholders suggested that a high level of OHL should be emphasised through *formal acknowledgement* by the state, such as state awards.

Another important aspect raised by stakeholders concern *support for key individuals*.

To actively involve individual healthcare professionals as well, the introduction of *targeted incentives* was proposed, such as the possibility of obtaining thematic certifications within the framework of continuing professional education, accompanied by the acquisition of recognised credits. In addition, the use of incentives is also suggested to stimulate the involvement of quality management teams in OHL measurement activities, encouraging them with effective motivational tools.

#### Integrating OHL into accreditation and external evaluation mechanisms

In order to achieve a sustained commitment to OHL accountability in health services, stakeholders recommended introducing specific *accreditation mechanisms*. They emphasised the importance of integrating OHL systematically and comprehensively into existing accreditation processes and making them an integral part of official evaluations. To support this approach, stakeholders have suggested the use of *certification systems* with different levels of recognition, such as gold, silver and bronze, valid for four-year. Similar systems have already been adopted, for example, in tobacco-free or child-friendly hospitals. Experience shows that organisations that achieve the highest levels of certification, such as the gold level, tend to meet the required standards, contributing to sustainable improvement over time.

To ensure that these standards are maintained and to support continuous improvement processes, it is also proposed that an *external figure* should oversee periodically—e.g., every six months—the monitoring of the organisations' progression, recording any changes and identifying new needs.

## Discussion

The analysis of stakeholders' responses was used to identify common macro-and micro-themes. In order to increase the likelihood of OHL being adopted by European healthcare organisations, managerial and leadership commitment, continuous staff training, OHL integration into organisational policies, and the active involvement of patients and the community were emphasised. Strategies to support the adoption of OHL assessments in healthcare organisations include clearly communicating their added value, securing endorsements and incentives from authorities and management, and providing practical, user‑friendly assessment tools. Hindering factors for OHL assessment included lack of awareness and understanding of OHL, lack of resources and management support as well as assessment tools perceived as complex. Supportive actors and concrete steps to obtain commitment to implementing OHL assessments were identified.

Macro- and micro-themes raised by stakeholders suggest that the adoption of OHL in healthcare system is not perceived as a set of isolated strategies of intervention, but rather a systematic organisational transformation requiring alignment between several elements. Taking together, all of them indicate that OHL needs a cultural and structural shift within healthcare organisations. In fact, the analysis reveals several aspects that could shape the adoption of OHL in healthcare systems. Stakeholders simultaneously called for strong formal mandates from leadership and management and for participatory approaches involving patients and communities, highlighting a balance between managerial authority and participatory processes. Stakeholders also oscillated between normative arguments (equity, patient-centredness) and managerial rationales (efficiency, benchmarking), suggesting that OHL is strategically framed to fit different institutional logics. Although stakeholders expressed strong support for OHL principles, they also highlighted work overload, lack of staff, limited awareness and insufficient resources, suggesting a gap between strategic aspiration and operational constraints. Moreover, voluntary engagement alone may be insufficient for a real adoption, in fact institutionalisation mechanism (e.g. standards, accreditation criteria, etc.) could be crucial for embedding OHL into routine organisational practice.

The SWOT analysis provide an interpretative framework [31] for synthesising stakeholders’ responses regarding the adoption of OHL interventions and OHL assessment in European healthcare organisations. Strengths, weaknesses, opportunities and threats were derived from the patterns emerging across the reported results and were used to support a critical and reflective interpretation of the findings and their practical implications. The SWOT analysis did not aim to systematically compare organisational differences, but to provide a structured lens for discussing common enabling factors and challenges identified starting from the responses provided by stakeholders.

The analysis highlights several strengths regarding the adoption of OHL by European healthcare organisations, strengths that could be considered solid foundations for its progressive development.

The first positive element concerns the presumed compatibility of OHL with existing systems, such as the quality system. This aspect suggests the possibility of integrating OHL interventions into already established processes, facilitating the involvement of key figures such as quality and risk managers. Those individuals are accustomed to working with assessment tools, and have the necessary expertise in process evaluation, compliance, and performance metrics to embed HL principles into organisational culture and practice. Kaper et al. [14] have also highlighted the importance of involving staff with knowledge of change strategies and quality improvement. However, the adoption of OHL into existing institutional processes could be supported if it is enhanced and sustained by government, organisational and partnership actions, which are able to improve the organisational capacity for delivering health-literate services [32]. A further strength is the existence of concrete examples of good practices. Indeed, some countries refer to successful cases, such as mhGAP and the Czech Action Plan [29, 30], which help enhance the credibility of the approach.

In addition, the adoption of simple but effective tools, such as Teach Back and Ask Me 3, further confirmed the practical applicability of OHL.

In this context, the inclusion and involvement of change champions has been found to contribute to the promotion of HL-friendly organisations in the long term [32]. From a theoretical point of view, some guidelines are already available to support the development of OHL within healthcare organisations, even if most have not been empirically tested in organisational settings, raising concerns about their practical effectiveness [33]. Farmanova et al. (2018) identified 20 HL guides facilitating the transition to organisational OHL, with the aim of mitigating HL gaps by simplifying healthcare, improving patient comprehension, strengthening support across all literacy levels, and offering evidence-based recommendations and quality improvement strategies [33].

Furthermore, standards, models and tools are used to assess OHL within healthcare organisations [3–35]. Over time, there seems to be increasing awareness of the need for specific training on HL at all levels, from clinic and administrative personnel to the patients themselves, as suggested by Peters (2008), Cifuentes et al. (2015) and Greaney et al. (2020) [36–38]. Effective communication, cultural competence, and the ability to assess HL are indeed essential competencies for all healthcare staff, given their impact on individual and population health and on the delivery of equitable care [38].

Another point of strength is the presence of initiatives already in use, such as infographics, signage, visual materials and explanatory videos. In this context, visual-based interventions, especially those using videos [39], represent a promising and practical approach for addressing limited HL, given their demonstrated effectiveness and broad applicability [40], enhancing patients’ comprehension of medications and treatments [41, 42].

Finally, the increasing recognition of the importance of patients’ and communities’ active involvement represents fertile ground for the development of OHL. The staff’s direct contact with needs and the feedback of users may indeed foster the development of more HL-oriented organisational practices [32–45].

The SWOT analysis also highlighted potential weaknesses in adopting OHL assessments in the healthcare organisations of the countries involved.

One of the main critical points concerns the excessive internal focus on operational and management problems and the persistence of top-down approaches in healthcare decisions. In this context, Adsul et al. (2017) emphasised, based on interviews, the importance attributed by stakeholders to communication strategies, as well as the use of external resources and technologies, as fundamental instruments for overcoming these critical weaknesses [46].

Another point of weakness is the lack, if not absence, of support from management, who, in some cases, may perceive the assessment of the OHL in case of negative an outcome as a reputational risk for organisations.

A cross-cutting weakness is that although standards and guidelines are available, they are either insufficient or not widely known enough to ensure the uniform adoption of OHL. This weakness is compounded by low familiarity with OHL concepts and practices among personnel, which can result, for example, in a lack of knowledge of tools that consequently may be perceived as complex, unsuitable or unlinked to the practice. In addition, perceptions of the practical benefits of an OHL assessment are limited.

Another weakness that has emerged is that training still predominantly focuses on clinical skills, giving little space to HL.

Other aspects that can weaken the implementation of OHL activities within organisations include the lack of a systematic collection of patients’ HL levels and the limited use of data that can emerge from listening to needs and analysing them.

Finally, the introduction of a systematic approach to OHL could be perceived as an additional workload unless it is an integral part of daily work.

The analysis revealed several elements that healthcare organisations may consider real opportunities for OHL development.

One such element is the increasing interest in patient-centered and HL-friendly approaches at the European level as evidenced by initiatives [47], such as the establishment of Communities of Practice.

Another concrete opportunity involves HL inclusion in regulatory frameworks and in national, European and healthcare plans [33], and this aspect could even allow the standardisation of assessment tools and promote further policies.

Moreover, from existing platforms, such as HelseNorge [28], it is possible to create centralised and validated national platforms to support the development of OHL in organisations.

Another opportunity is increasing healthcare digitalisation. In healthcare organisations, the use of digital tools aimed at supporting communication with patients and follow-ups is starting to spread, and the use of digital innovations such as the use of QR codes, video tutorials and online tools may simplify the diffusion and use of OHL assessment tools.

From a political and institutional point of view, an increasing incentive emerges for the active participation of citizens, which may also represent a starting point for the potential co-design of projects and digital services involving users.

Finally, the promotion of a systematic approach to OHL within healthcare organisations become a hallmark of benchmarking and competitiveness.

The SWOT analysis underlines threats to the promotion and systematic implementation of OHL within healthcare organisations. One threat is represented by ineffective communication, aimed at both staff and patients, which could compromise the understanding, acceptance and effectiveness of initiatives linked to HL.

Another negative aspect is work overload. In fact, the introduction of new tools may be experienced as an additional administrative and clinical burden, especially in contexts that are already under pressure [46].

Importantly, the needs of healthcare organisations may vary depending on the local and geopolitical context in which they are located: for example, the Ukrainian healthcare context is influenced by the ongoing war conflict. Moreover, healthcare contexts are not homogeneous, and differences in structure, resources and skills may be difficult elements for the uniform adoption of OHL.

In general, healthcare organisations are described as resistant to organisational change, characterised by a reluctance to embrace new tools and approaches.

Another relevant threat concerns the difficulties in involving individuals who face language, cultural, or digital barriers, which can restrict their access to information and participation in healthcare processes in general [48]. Jaeger et al. (2019), for example, highlighted the significant challenge posed by language barriers in primary care settings, emphasising the importance of improved access to professional interpreting services as a key strategy to ensure equitable communication and support [49].

A further threat may arise from the complex tools for the assessment of OHL.

Finally, political and institutional discontinuity, as well as changes in the government, may result in the interruption of long-term support or delay the integration and development of OHL within healthcare organisations.

Having an overview of the factors hindering and facilitating OHL in healthcare, identified by stakeholders from different countries, could be useful for developing more specific strategies and interventions to support the adoption of OHL within healthcare. Identifying these factors reveal implementation pathways, such as organisation-wide approaches. The stakeholders’ perspectives suggest that strategies to promote OHL in healthcare systems should move beyond isolated initiatives, therefore multi-level alignment is required: for example, at the governance level, OHL should be anchored in accreditation systems and regulatory framework; at the organisational level, assessment processes should be integrated into existing quality management systems; at the workforce level, continuous and context-sensitive training is needed to build competences and reduced perceived burden; at the community level, participatory processes should ensure responsiveness to diverse needs. This informs, for example, evidence-based toolkits, training standardisation and policy integration, strengthening the causal links between OHL interventions.

This study has limitations that should be acknowledged. First, the baseline levels of awareness and familiarity with OHL among stakeholders differed, potentially influencing the depth and framing of their responses. Second, data collection procedures were not fully consistent across countries. Each national research team selected its preferred mode of interview administration. While this approach may have enhanced intra-group reliability by allowing procedures to be adapted to local contexts, it may also have increased inter-group reliability, potentially affecting the comparability of responses across countries. Third, the respective national research teams conducted the translations of interview questions into English. Translators ensured accuracy by referring back to the original interview notes or summaries whenever clarification was needed. While this process aimed to preserve key nuances, stakeholder expressions and contextual meanings, the lack of independent external verification may have meant that subtle linguistic and cultural nuances were not fully captured in the English versions of the data.

Future research could explore whether the adoption of different interview modalities reflects underlying cultural or contextual factors. In particular, it would be valuable to examine whether such methodological choices are influenced by cultural norms, research traditions or the systemic characteristics of national health systems. Furthermore, quantitative analyses could be conducted to determine whether variations in response patterns are associated with differences in data collection methods. This would clarify the extent to which methodological variability influences intra- and inter-group differences in findings.

## Conclusions

Although OHL plays a critical role in ensuring equitable, effective, and patient-centered care, it remains insufficiently recognised and adopted across many European healthcare systems. By engaging relevant stakeholders from six European countries—Austria, Hungary, France, Italy, Norway, and Ukraine—this study provides a rich, cross-national perspective on OHL and the conditions necessary for its successful adoption and promotion with a specific focus on the implementation of OHL assessments.

The analysis revealed nuanced macro- and micro-themes across six key areas, emphasizing that improving OHL is not merely a technical task but a strategic and cultural shift. Strong leadership and managerial commitment emerged as foundational, alongside the need for continuous staff training in communication, empathy, and information accessibility. Integrating OHL into organisational policies and involving patients and communities are essential steps toward embedding OHL into everyday practice.

Stakeholders highlighted that OHL assessments must demonstrate tangible benefits, such as enhanced care quality and cost reductions, to gain traction. However, several barriers—high workloads, resistance to change, complex tools, and limited resources—must be addressed through simplification strategies and targeted incentives.

Ultimately, the successful adoption of OHL depends on leadership support, alignment with quality improvement strategies, and sustained staff training. Anchoring OHL in regulatory frameworks and establishing mechanisms for ongoing monitoring will be key to ensuring long-term commitment and impact.

Considering the various facilitating and hindering factors provides a useful basis for developing strategies to enhance OHL and promote the adoption of specific interventions within healthcare organisations. Stakeholders' perspectives, informed by their expertise in HL and direct knowledge of the context, could be therefore a valuable resource for defining concrete, sustainable paths towards the adoption of OHL in European healthcare organisations.

## Supplementary Information

Supplementary Material 1.

## Data Availability

The datasets generated, used and analysed during the interviews are or will be available from the corresponding author upon reasonable request.

## Abbreviations

HL: Health Literacy
HLHO-10: Health-Literate Healthcare Organisation 10 item questionnaire
JA PreventNCD: Joint Action Prevent Non-Communicable Diseases
MhGAP: Mental Health Gap Action Programme
OHL: Organisational Health Literacy
OHL-Hos: International Self-Assessment Tool for Organizational Health Literacy (Responsiveness) of Hospitals
OHL-PHC: International Self-Assessment Tool for Organizational Health Literacy in Primary Health Care Services
