# Supplementary material for: Organisational health literacy in healthcare in Europe: a content analysis to explore stakeholders perspectives for supporting the adoption of effective strategies and interventions
Source: Arch Public Health. 2026 May 16;84:151. doi: 10.1186/s13690-026-01956-6 (PMC13352983; doi:10.1186/s13690-026-01956-6)
Supplement: Supplementary file 1 — Supplementary Material 1. [file 13690_2026_1956_MOESM1_ESM.docx]

**Table 1:** Sociodemographic characteristics and professional backgrounds of stakeholders and experts by country.

| **Stakeholder ID** | **Gender** | **Age** | **Professional background/expertise** |
| --- | --- | --- | --- |
| AT_EXP_01 | Female | N/A | Organisational health literacy in hospitals |
| AT_EXP_02 | Female | N/A | Organisational health literacy |
| AT_EXP_03 | Female | N/A | Health literacy in hospitals; (Other) staff in health care services in leading roles |
| AT_EXP_04 | Female | N/A | Policy in the field of health care/on health issues; Organisational health literacy; Health literacy |
| AT_EXP_05 | Female | N/A | Health literacy; (Other) staff in health care services in leading roles |
| AT_EXP_06 | Male | N/A | Management (upper management, quality management) of health care organisations |
| FR_EXP_01 | Female | N/A | Policy in the field of health care/on health issues; Organisational health literacy; Health literacy; NCDs and/or cancer prevention |
| FR_EXP_02 | Female | N/A | Health literacy; NCDs and/or cancer prevention |
| FR_EXP_03 | Male | N/A | Health literacy; NCDs and/or cancer prevention; (Other) staff in health care services in leading roles |
| FR_EXP_04 | Male | N/A | Policy in the field of health care/on health issues; NCDs and/or cancer prevention; Management (upper management, quality management) of health care organisations; Representatives from professional associations |
| FR_EXP_05 | Female | N/A | Policy in the field of health care/on health issues; NCDs and/or cancer prevention; Management (upper management, quality management) of health care organisations; |
| FR_EXP_06 | Male | N/A | Policy in the field of health care/on health issues; Health literacy; NCDs and/or cancer prevention; (Other) staff in health care services in leading roles |
| FR_EXP_07 | Male | N/A | Policy in the field of health care/on health issues; Health literacy; NCDs and/or cancer prevention; (Other) staff in health care services in leading roles |
| FR_EXP_08 | Female | N/A | Policy in the field of health care/on health issues; Health literacy; NCDs and/or cancer prevention; (Other) staff in health care services in leading roles |
| FR_EXP_09 | Female | N/A | Policy in the field of health care/on health issues; Organisational health literacy; Health literacy; NCDs and/or cancer prevention |
| FR_EXP_10 | Female | N/A | Organisational health literacy; Health literacy; NCDs and/or cancer prevention |
| HU_EXP_01 | Female | N/A | Organisational health literacy; Health literacy; NCDs and/or cancer prevention |
| HU_EXP_02 | Female | N/A | Organisational health literacy; Health literacy; NCDs and/or cancer prevention |
| HU_EXP_03 | Female | N/A | Health literacy; NCDs and/or cancer prevention |
| HU_EXP_04 | Female | N/A | Health literacy; NCDs and/or cancer prevention |
| HU_EXP_05 | Female | N/A | Management (upper management, quality management) of health care organisations |
| HU_EXP_06 | Female | N/A | Management (upper management, quality management) of health care organisations |
| HU_EXP_07 | Female | N/A | Other) staff in health care services in leading roles |
| HU_EXP_08 | Female | N/A | Other) staff in health care services in leading roles |
| HU_EXP_09 | Male | N/A | Other) staff in health care services in leading roles |
| HU_EXP_10 | Female | N/A | Organisational health literacy; Health literacy; NCDs and/or cancer prevention |
| IT_EXP_01 | Male | 67 | Medical doctor, Hospital Direction |
| IT_EXP_02 | Female | 52 | Medical Doctor, Head of Clinician Hospital Unit |
| IT_EXP_03 | Female | 55 | Sociologist, Prevention |
| IT_ EXP_04 | Male | 58 | Medical Doctor, Clinical Government and Risk Management |
| IT_EXP_05 | Female | 64 | Medical Doctor, Prevention |
| IT_EXP_06 | Male | 59 | Medical Doctor, Hospital Direction |
| IT_EXP_07 | Male | 42 | Medical Doctor, Prevention |
| IT_EXP_08 | Male | 59 | Medical Doctor, Prevention |
| IT_EXP_09 | Male | 75 | Retired Medical Doctor, Patients' representative |
| IT_EXP_10 | Male | 43 | Medical Doctor, Prevention |
| IT_EXP_11 | Male | 64 | Statistic, National Institute of Health |
| NO_EXP_01 | N/A | N/A | Health literacy; NCDs and/or cancer prevention; Management/staff who already participated in OHL assessment; Management (upper management, quality management) of health care organisations; Patients representatives; Representatives from specialist health care; Representatives from primary health care |
| NO_EXP_02 | N/A | N/A | Policy in the field of health care/on health issues; Health literacy; NCDs and/or cancer prevention; Management/staff who rejected participated in OHL assessment; Patient representatives; Representatives from specialist health care |
| NO_EXP_03 | N/A | N/A | Health literacy; NCDs and/or cancer prevention; Management/staff who already participated in OHL assessment; Representatives from specialist health care; Representatives from primary health care |
| NO_EXP_04 | N/A | N/A | Health literacy; Management (upper management, quality management) of health care organisations |
| NO_EXP_05 | N/A | N/A | Health literacy; NCDs and/or cancer prevention; Management/staff who rejected participated in OHL assessment; Management (upper management, quality management) of health care organisations; Representatives from professional associations; Representatives from specialist health care; Representatives from primary health care |
| NO_EXP_06 | N/A | N/A | Health literacy; Management (upper management, quality management) of health care organisations; Representatives from primary health care |
| NO_EXP_07 | N/A | N/A | Health literacy; NCDs and/or cancer prevention; Management (upper management, quality management) of health care organisations; Representatives from specialist health care; Representatives from primary health care |
| NO_EXP_08 | N/A | N/A | Policy in the field of health care/on health issues; Health literacy; Management (upper management, quality management) of health care organisations; Patients representatives; Representatives from specialist health care; Representatives from primary health care |
| UA_EXP_02 | Male | 33 | Policy makers in the field of health care/on health issues |
| UA_EXP_04 | Female | 34 | Policy makers in the field of health care/on health issues |
| UA_EXP_05 | Male | 49 | Management (upper management, quality management) of health care organisations |
| UA_EXP_07 | Female | 52 | Patient representatives |
| UA_EXP_03 | Female | 55 | Experts on OHL and NCD and/or cancer prevention |
| UA_EXP_01 | Male | 62 | Staff in health care services in leading roles (who have some influence on organisational structures and change) |
| UA_EXP_06 | Female | 65 | Management (upper management, quality management) of health care organisations |
|  |  |  |  |
